# Editorial: Airway Surgery in Children

**DOI:** 10.3389/fped.2022.865159

**Published:** 2022-03-16

**Authors:** Michele Torre, Rebecca Maunsell, Patricio Varela

**Affiliations:** ^1^Pediatric Thoracic and Airway Surgery, IRCCS Giannina Gaslini, Genoa, Italy; ^2^Pediatric Otolaryngology Division, State University of Campinas (UNICAMP), Campinas, Brazil; ^3^Pediatric Surgery, Calvo Mackenna Children Hospital and Clinica Alemana, Santiago, Chile

**Keywords:** airway, trachea–abnormalities, pediatrics, surgery, airway stenosis

Airway surgery in children is a challenging field, requiring highly competent and organized surgeons and airway teams. An increasing number of pediatric airway teams have been established all over the world. The article “*Teamwork in Airway Surgery*” reported by Elliott et al. describes the principles, obstacles, and solutions that inspired one of the first and busiest airway teams in the world (based in London). Acknowledging the local history, the theory and mindset behind the conception of a tracheal team, how to deal with practical and ideological difficulties, and future perspectives, is very inspiring for anyone who is dedicating efforts to the care of pediatric airway patients. Our recently published (1) experience is quite similar and has been directly inspired by the London tracheal team.

The first approach to the patient with airway symptoms through endoscopic evaluation is in most cases crucial to define an early and precise diagnosis. “*Pharyngomalacia in neonates: the missed issue*” by Moslehi et al. describes a condition often misdiagnosed, causing noisy breathing or more severe symptoms, and provides useful information on how to perform precise endoscopic evaluation, establishing the severity of the condition, and offering possible treatment options. It is an interesting manuscript as it addresses an unacknowledged and probably underdiagnosed cause of airway obstruction in neonates, problems for differential diagnosis, and choices of treatment.

In “*Endoscopic, preoperative assessment, classification of stenosis, decision-making*” the authors underline the importance of a step-by-step rigorous endoscopic evaluation, including various practical maneuvers and tips that lead to a precise classification of the stenosis and consequently the most appropriate treatment options (Filauro et al.). Among the available classifications, the authors propose the new European Laryngeal Classification (2), which is a useful instrument to tailor the best treatment modality for each patient. In our opinion, the value of a well**-**conducted pre-operative endoscopic evaluation cannot be overestimated and is the first step to providing the correct surgical approach and outcome.

The most important message of “*Ongoing laryngeal stenosis: conservative management and alternatives to tracheostomy*” by Schweiger and Manica is to emphasize that a proactive endoscopic treatment is effective in most cases of ongoing laryngeal stenosis, avoiding tracheostomy in the majority of patients. While in the past most of the patients who could not be weaned off ventilation through an endotracheal tube were submitted to a tracheostomy. In recent years, with proper repeated endoscopic treatment and follow-up, a tracheostomy should be considered only as the last resort. However, the best treatment modality (balloon vs. bougies) and the role of adjuvant treatments (steroids, mitomycin, topic ointments, pump inhibitors) have still to be determined. It is also important to recognize that dilatation can be repeated, but if the patient requires more than 3 or 4 dilatations another treatment option should be considered.

In “*Surgical management of anterior glottic webs*,” Kuo and Rutter provide an article with great technical detail and useful illustrations, describing how to treat laryngeal webs, which are rare congenital anomalies often associated with 22q11.2 deletions, encompassing different degrees of severity, and requiring different approaches. The less experienced and confident readers with these challenging patients will take advantage of the large experience of the Cincinnati Airway team, described in this manuscript.

The treatment of bilateral vocal cord palsy remains a very controversial issue. Trozzi et al. in “*Surgical options for pediatric vocal cord palsy: state of the art*” present a comprehensive review of the literature describing several surgical techniques proposed over the last decades and underline the importance of pursuing, if possible, two main goals or principles: avoiding tracheostomy and being very conservative when it comes to preserving voice quality both avoiding permanent scarring to the vocal cords and/or oversizing the posterior glottis. These concepts are especially valuable for our pediatric patients.

Another very controversial and challenging topic is recurrent papillomatosis. The manuscript “*Airway Papillomatosis: new treatments for an old challenge*” by Kumar and Preciado provides new insights on innovative approaches as novel instrumentation for endoscopic removal of papillomas or even alternatives to it. Among the novel adjuvant therapies described, systemic bevacizumab is a promising treatment that could probably represent an alternative to the surgery itself. In particular, there are reports on the efficacy of systemic bevacizumab in treating aggressive papillomatosis not responsive to other treatments. The potential appeal is enhanced by the fact that bevacizumab seems to be more effective on tracheobronchial localizations, which are more difficult to treat and surgically assess and tend to significantly impact patients' morbidity and mortality. Prospective studies in larger series using standardized protocols and long follow-up have to be conducted to validate and make this new treatment option more widespread.

Kamran and Jennings in “*Tracheomalacia and tracheobronchomalacia in Pediatrics: an overview of evaluation, medical management, and surgical treatment*” present a comprehensive description of the anatomy, clinical symptoms, medical, and surgical treatment of tracheobronchomalacia, a challenging condition, often under- or misdiagnosed in pediatrics. The global approach to these patients includes a three phase bronchoscopic evaluation and a dynamic angio CT scan to study airway/vascular conflicts. The surgical treatment should be individualized. Particularly in those patients, presenting intrusion of the posterior wall of the trachea, posterior tracheobronchopexy, recently described by Boston group (3), which seems to be a better option than the classical approach through an aortopexy (4). As for other airway conditions, a multidisciplinary approach, taking into account feeding and breathing patterns and associated symptoms both during the day and during sleep that may impact a child's general growth and development, is the only way to offer the best treatment for the individual patient affected with tracheomalacia.

The last four papers of this issue describe clinical cases. In “*The important role of endoscopy in management of pediatric pseudomembranous necrotizing tracheitis*,” the authors propose the use of flexible endoscopy in children presenting acute onset of respiratory obstruction (Wu X. et al.). Although in this condition tracheal fragility could be a contraindication to endoscopy, the authors present a favorable outcome in two patients, in which the procedure provided an early diagnosis and consequently prompted mechanical debridement.

The importance of endoscopy, associated with CT scan, for differential diagnosis in an infant presenting with wheezing, is stressed by the group of Cutrera, who in “*Recurrent wheezing in pre-school age: not only airway reactivity!*” presented a case of the surgical removal of a mediastinal bronchogenic cyst (Roversi et al.). In our experience, there are a wide range of pathologies that must be considered and that have been diagnosed in children treated for a long time for bronchial hyper-reactivity or asthma, both primary conditions such as tumors, malacia, congenital laryngotracheal stenosis, and secondary vascular or mass compression. The message of this report is that the threshold for more invasive investigation as endoscopy and CT scan should not be too high in symptomatic and not responding patients.

Primary tracheobronchial tumors in children are rare, and surgery is the only therapeutic tool in many cases (5), for which open or endoscopic approaches can be appropriate options. In the case presented by Wu L. et al. (“*Case Report: Resection of Giant Endotracheal Hamartoma by Electrosurgical Snaring via Fi beroptic Bronchoscopy in a 9-Year-Old Boy*”) a tracheal hamartoma was successfully removed endoscopically using interventional bronchoscopy. The authors wisely reinforce the importance of a multidisciplinary approach to the patient, for whom different surgical approaches had been discussed. Another example of a multidisciplinary approach is described by Bing et al., who wrote “*Congenital Bronchobiliary Fistula: A Case Report and Literature Review*”. A rare congenital fistula was diagnosed through fistulography performed during a bronchoscopy and successfully removed thoracoscopically. In these rare anomalies of the airway, such as the one presented here, bronchography associated with bronchoscopy can still play an important role, as demonstrated in the literature and our experience (6–8).

In conclusion, “Airway surgery in children” comprehends various congenital and acquired airway conditions, some more others less common, but all of them represent significant challenges for the airway teams. The present issue provides many important messages that can be summarized in the use of a multidisciplinary approach with rigorous and rational diagnostic work-up to establish prompt and appropriate treatment tailored to each patient by an experienced surgeon open to innovative approaches and using age appropriate tools for young patients.

## Author Contributions

All authors listed have made a substantial, direct, and intellectual contribution to the work and approved it for publication.

## Conflict of Interest

The authors declare that the research was conducted in the absence of any commercial or financial relationships that could be construed as a potential conflict of interest.

## Publisher's Note

All claims expressed in this article are solely those of the authors and do not necessarily represent those of their affiliated organizations, or those of the publisher, the editors and the reviewers. Any product that may be evaluated in this article, or claim that may be made by its manufacturer, is not guaranteed or endorsed by the publisher.
